# Ten-year outcomes of patients who developed persistent azoospermia following chemotherapy associated with different oncological diagnoses: A retrospective cohort study from a different perspective

**DOI:** 10.55730/1300-0144.5373

**Published:** 2022-03-12

**Authors:** Ahmet ŞALVARCI, Ali Sami GÜRBÜZ, Mehmet BALASAR

**Affiliations:** 1 Department of Urology, Novafertile and Medicana Hospital IVF Centers, Medicana Hospital Affiliated with KTO Medical Faculty, Konya, Turkey; 2 Department ofObstetrics and Gynecology, Novafertile IVF Centers, Medicana Hospital Affiliated with KTO Medical Faculty, Konya, Turkey; 3 Department of Urology, Meram Faculty of Medicine, University of Necmettin Erbakan, Konya, Turkey

**Keywords:** Azoospermia, chemotherapy, m-TESE, SDFI, ROS, male, infertility

## Abstract

**Background/aim:**

This study evaluated the treatment procedures for chemotherapy (CT)-induced persistent azoospermia and their outcomes from a different perspective.

**Materials and methods:**

In 63 patients (mean age: 30.16 ± 4.91 years) who had undergone CT 11 ± 5 years earlier, the semen volume, gonadotropins level, FSH level, genetics, micro-testicular sperm extraction (m-TESE) result, sperm DNA fragmentation index (SDFI), semen reactive oxidative stress (ROS) rate, duration of embryonic development, and pregnancy and baby take-home rates were examined. The correlations between the ROS rates and the SDFIs, m-TESE results, sperm motility, pathology scores, time-lapses, and baby take-home rates were evaluated.

**Results:**

The semen volumes were 3.5 ± 1.1/ml. The FSH level following CT was 17.87 ± 5.80 mIU/ml. A sperm rate of 34.9% was found from the m-TESE result. The mean SDFI and ROS rate were 4 (<15–30>) and 1.29 ± 0.51, respectively. The time-lapse was calculated as 5h. Pregnancy and live birth were achieved at 20.63% and 12.7%, respectively. In the patients with a low ROS (≤1.42) and SDFI (≤15), the m-TESE success rate was high, the FSH value was low, the pathological score and fertilization rate were elevated, the embryonic cleavage period was normal, and the pregnancy and baby take-home rates were high.

**Conclusion:**

The sperms may be detected using m-TESE in patients who develop persistent azoospermia associated with CT due to different oncological diagnoses. Our study revealed that a low FSH value and normal ejaculatory ROS rates are positive predictive factors of sperm detection before m-TESE. The motility of the sperms detected after m-TESE and normal SDFI rates were found to be positive predictive criteria of high fertilization, good embryonic cleavage, pregnancy, and live birth.

## 1. Introduction

After cardiovascular diseases, cancer is one of the most commonly diagnosed diseases worldwide. According to the World Health Organization (WHO), 9.6 million people died due to cancer in 2018 [1]. Early diagnosis, new-generation chemotherapy (CT) agents, and modifications in treatment protocols have had positive impacts on life expectancy [2]. However, these treatments are known to harm the spermatogenetic activities of the testes and many other organs [2].

Approximately 7% of men worldwide are infertile [3,4]. Primary infertility refers to the failure of spouses to achieve a clinical pregnancy despite 12 months of unprotected sexual intercourse. Secondary infertility refers to the failure of spouses to have a second child. Men are solely responsible for 20%–30% of primary infertility cases and for about 50% of primary and secondary infertility cases combined [3,4]. Male infertility has many causes, such as genetic factors, varicocele, hormonal dysfunctions, obstructive causes, testicular trauma, undescended testis, unhealed testicular infections, mumps, and cancer [3,4].

Reactive oxygen species (ROS) are significant factors of male infertility because they decrease sperm production and damage the produced sperm [3,4]. CT and radiation therapy or radiotherapy (RT) lead to these outcomes because they generate ROS in the short and long terms [4]. They severely damage the sperm DNA, mitochondria, and nuclei [4] and are thus transiently or even permanently toxic for sperms and sperm stem cells [5,6]. Sperm stem cells are fast-growing cells that are particularly highly sensitive to CT. They begin to die within two to three months after CT. Consequently, permanent azoospermia may develop [5, 6]. In our clinical practice, we have measured the damage in the sperms and semen of our azoospermia patients through their ROS and sperm DNA fragmentation index (SDFI) rates [4]. There are two ways to preserve fertility in these patients: by freezing sperms or testicular tissues before treatment (called sperm freezing or sperm cryopreservation) or by retrieving sperms via micro-testicular sperm extraction (m-TESE) after treatment. Unfortunately, it may not be possible to retrieve sperms with m-TESE following CT [7]. Therefore, sperm cryopreservation is the safest method [7]. In this study, the clinical outcomes of CT-induced persistent azoospermia patients were evaluated, and the laboratory results of treatment procedures, including the surgical, fertilization, pregnancy, and live birth rates, were assessed in terms of their correlation with the patients’ measured ROS and SDFI rates.

## 2. Materials and methods

The files were reviewed retrospectively upon the approval of the ethics committee of the school of medicine of Necmettin Erbakan University, through its decision no. 2019/2107 adopted in its Session 1 on 01/10/2019. A total of 63 patients had been screened between 2008 and 2018. Their semen analyses were checked twice via pellet control (WHO, 2010). Their follicle-stimulating hormone (FSH), luteinizing hormone (LH), total testosterone (TT), and prolactin (PRL) were checked. Their urine and ejaculate were assessed microbiologically. The FSH values before CT and after m-TESE were recorded. The karyotypes and Y chromosome microdeletions were checked. The SDFI rates were detected via (terminal deoxynucleotidyl transferase dUTP nick end labeling) TUNEL assay. SDFI values of **<**15, 15–30, and **>**30 were regarded as normal, suspicious, and positive, respectively [8]. Between 2016 and 2018, the ROS levels in fresh neat semen of the patients were measured using a chemiluminescence assay in a single-tube luminometer (MiOXSYS^®^ system) [9]. The ROS value of <1.42 mV/10^6^ sperms/mL in the semen was regarded as normal [9]. The patients underwent m-TESE once or twice. M-TESE was applied with a surgical light microscope with a 25 × 13.5-fold magnification power under anesthesia. The testis tissue pathologies were evaluated using the Johnsen score (footnote, Table 1, cut-off score of ≥4 in the presence of sperms). The embryos were followed up with a time-lapse (Unisense Fertilitech^®^) [10]. The sperm tissues and embryos were preserved after intracytoplasmic sperm injection (ICSI). The pregnancy and baby take-home rates were followed up. The m-TESE and FSH rates, pathologies, fertilized oocyte counts, pregnancy rates, sperm motility/nonmotility rates, and embryonic cleavage period under a time-lapse were evaluated individually according to their ROS positivity/negativity and SDFI <15/>15 levels (suspicious + positive group) to identify whether they were a criterion. We excluded other factors that could affect the ROS and SDFI rates, and we tried to demonstrate the impact of CT on spermatogenetic activity regardless of whether it was subjective.

### 2.1. Statistical analysis

In the descriptive data statistics, the values of mean, standard deviation, median lowest, highest, frequency, and ratios were utilized. The distribution of the variables was measured by the Kolmogorov Smirnov test. The independent samples T-test and Mann-Whitney U test were used in the analysis of quantitative independent data. The Wilcoxon test was utilized in the analysis of dependent quantitative data. The chi-square test, or in case of failure to fulfill the necessary conditions for the chi-square test, the Fisher’s exact test was used in the analysis of independent quantitative data. The SPSS 22.0 program was utilized in the analyses.

## 3. Results

The mean number of years following CT was 9.9 (6–16 y). The patients who were admitted to the hospital for infertility treatment had not been receiving any tumor-related treatment. Thirteen patients had undergone bone marrow transplantation for acute lymphocytic leukemia (ALL), three patients; for acute myeloid leukemia (AML), three patients; for non-Hodgkin’s lymphoma (NHL), four patients; and for Hodgkin’s lymphoma (HL), one patient. While testicular cancers were observed most frequently (in 32/63 patients (50.7%)), chronic myeloid leukemia (CML), Ewing sarcoma, and Wilms’ tumor were detected less frequently (in 1/63 patient (1.6%) for each condition) (Table 1). The examinations of the patients revealed that eight of them had undergone varicocelectomy before CT, and seven had varicocelectomy following CT. BEP (Bleomycin Etoposide Cisplatin) was administered for testicular volumes at 3-week intervals with a median of 3 cycles, while radiotherapy was administered to 6 patients with seminoma. The ALL patients received one cure each of vincristine, steroid, L-asparaginase, and cyclophosphamide, and the AML patients received three cures each of daunorubicin, idarubicin, and mitoxantrone (on three consecutive days). The CML patient received one cure each of splenic irradiation, busulfan, hydroxyurea, interferon alfa, imatinib, and dasatinib. The HL patient received three cures of MOPP (mechlorethamine hydrochloride, vincristine sulfate, procarbazine hydrochloride, and prednisone) while RT was applied to the lesion areas. The NHL patients received R-CHOP (rituximab, cyclophosphamide, doxorubicin, vincristine, and prednisolone) and then underwent head neck RT. The rhabdomyosarcoma patient received vincristine and actinomycin and then underwent RT. The Ewing sarcoma patient received vincristine, adriamycin, actinomycin-D, and cyclophosphamide and then underwent RT. The osteosarcoma patient received adriamycin, methotrexate, cisplatin, ifosfamide + mesna, and etoposide and then underwent RT. The patient with Wilms tumor received actinomycin D, vincristine, and doxorubicin and then underwent RT. The CT and RT protocols of nine patients with testicular tumors were renewed, and four patients underwent retroperitoneal lymphatic gland dissection. In three NHL patients and two HL patients, the CT/RT cures were repeated after their cancer returned following remission. Types 1 and 2 diabetes mellitus were diagnosed in two seminoma patients, three nonseminoma patients, one HL patient, and two NHL patients. No patient was treated for spermatogenetic activation before and during m-TESE. Hypertension was detected in one HL and three NHL patients. All the patients applied while they were experiencing persistent azoospermia. None of their sperms had been frozen previously. They reported that they had not been informed regarding this matter. All the patients had the karyotype 46XY. Partial azoospermia factor C (AZFc) deletion was identified in one patient. The semen analyses revealed pellet negativity with azoospermia with an average volume of 3.3 cc (3.26 ± 1.13). Sperms were detected at a rate of 34.9% in a total of 22/63 patients in their first and second m-TESE procedures (Table 2). While the sperm retrieval rate with m-TESE was highest in 14/32 (43.7%) of the patients with testicular cancer, sperms were detected in 2/8 (25%) of the ALL patients, in 1/3 (33%) of the patients with osteosarcoma, in 1/3 (33%) of the AML patients, in 1/4 (25%) of the HL patients, and in 3/8 (37.5%) of the NHL patients. No sperm was found in the patients with Wilms’ tumor, soft tissue Ewing sarcoma, CML, and rhabdomyosarcoma. The average m-TESE duration was 102 min (25–161 min). Pregnancy was achieved after the first and second ICSI at a rate of 59%. The pregnant women’s baby take-home rate was 12.7%. The pathological mean Johnsen (JHS) score in the m-TESE positive (+) group was 6, and the mean rate was 2 in the m-TESE negative (−) group. The m-TESE was repeated in 15 patients whose first m-TESE (−) resulted in the detection of sperms in six patients.

Nonmotile sperms were detected in two patients in the repeat m-TESE during their quarterly/biannual follow-ups. However, no embryo development was seen in one patient after ICSI, and no pregnancy was achieved in the other patient.

In the m-TESE (+) group, the SDFI was <15 in 13 patients, ≥15 in two patients, and >30 in two patients. The ROS test resulted in a normal rate of <1.42 in 12 patients, and in a high rate of ≥1.42 in six patients. Embryos were followed up with a time-lapse after ICSI. In practice, a blastomere separation period of <6 h is regarded as normal [9]. While the mean blastomere separation period in all the patients was 5 h, the mean separation period in the 13 patients in the pregnancy group was >11 h on average (Table 2).

### 3.1. Statistical results in the m-TESE (+) and (−) groups

The ages of the patients and their spouses and in the marriage durations; smoking rates; m-TESE periods; FSH, LH, TT, and PRL values before CT; and m-TESE and ROS test results did not differ significantly (p > 0.05) between the m-TESE (−) and m-TESE (+) groups. The FSH value before the m-TESE increased significantly (p < 0.05) after CT in the m-TESE (−) group. Likewise, the FSH value before the m-TESE increased significantly (p < 0.05) after CT in the m-TESE (+) group. The semen volumes in the m-TESE (+) group were significantly higher (p < 0.05) than those in the m-TESE (−) group in the testicular volume value at the right and left scrotal color doppler (Table 3).

### 3.2. Statistical results according to the ROS (+) and (−) values

In the ROS (−) group, the m-TESE positivity was significantly higher and the FSH value was significantly lower than in the ROS (+) group (p < 0.05). In both the ROS (−) and ROS (+) groups, no significant difference (p > 0.05) was observed in the m-TESE success rate, Jhs score, fertilized oocyte count, pregnancy rate, distribution rate of motile and immotile sperms, and <5h time-lapse cleavage values (Table 4).

### 3.3. Statistical results according to the SDFI levels of <15 and ≥15

In the group with an SDFI of <15, the m-TESE positivity, JHS score, and fertilized oocyte count were significantly higher (p < 0.05) than those in the group with an SDFI of >15. In the group with an SDFI of <15, the FSH value was significantly lower (p < 0.05) than that in the group with an SDFI of ≥15. In the groups with an SDFI of <15 and an SDFI of ≥15, insignificant differences (p > 0.05) in the ICSI-total pregnancy rates, motility/nonmotility sperm distributions, and <5h time lapses were observed (Table 4).

In five patients with an FSH value of <12 mIU/L, a ROS rate of <1.42 mV/106 sperms/mL, an SDFI rate of <15, and an embryonic cleavage period of <6 h at the time-lapse, fertilization occurred in 4/5 (80%) patients, and the baby take-home rate was 4/5 (80%) patients, which were significantly higher than in the general group (p < 0.05). In four patients with an FSH value of >25 mIU/L, a ROS rate of >1.42 mV/106 sperms/mL, an SDFI rate of >25, and an embryonic cleavage period of >12 h at the time-lapse, the fertilization rate was 1/4 (25%) patients, and the pregnancy rate was 1/4 (25%) patients, but the pregnancy with an intrauterine ex fetus was terminated. The rates in this group were significantly lower than those in the general group (p < 0.05) (Table 4). The Jhs score was >6 in the patients with a low FSH value, a normal ROS rate, and no sperm detected at the m-TESE. For the patients with high FSH, ROS, and SDFI rates, the JHS score was <3. In the group that brought home a child, the sperms of the patients were motile. The same results were seen in five patients from whom sperms were obtained at the m-TESE, whose FSH rate was low, and whose ROS and SDFI rates were normal (Table 4).

Testicular volumes before and after chemotherapy, semen analysis, hormone, and reactive oxidative stress mean values according to cancer diagnosis are shown in Table 5.

## 4. Discussion

As stated previously, both cancer and cancer treatments such as CT, RT, and biologic therapies harm male fertility by disrupting sperm production. The most frequently used strategies to address this issue are sperm cryopreservation and sperm banking before cancer treatment. However, they are not yet widely used. One reason for this is that patients are not sufficiently informed about them, as were our patients [11,12]. In many patients who had undergone CT due to various types of cancer, sperms may still be detected in their semen within one to six years after their CT. However, azoospermia becomes permanent in one-third of cases [11,12]. In a study with participants similar to those in this study but older, 67 couples aged 24–44 years who were diagnosed with testicular cancer or lymphoma received adjuvant treatment with CT and/or RT following BEP and MOPP treatment. A permanent sperm loss of 57% was noted in the group that did not undergo sperm freezing, despite having had a child after 82 ICSI and 14 ICSI-frozen embryo replacement procedures [12]. In this study, persistent azoospermia was found at a higher rate, in 41/63 (65%) patients. Although the negative impacts of different chemotherapeutic agents on spermatogenetic activity have been disclosed, unfortunately, whom among patients will or will not be impacted is still uncertain [7, 13]. In the study conducted for this purpose, sperm freezing was found necessary in the cases of 12 patients who received BEP/MOPP CT/RT for testicular cancer and HL. Although sufficient sperms were detected for ICSI in some patients after treatment, permanent azoospermia still developed; and even after m-TESE, no sperm could not be found in some patients [13]. It is recommended that relevant measures be adopted for fertility as early as possible before initiating the treatment [7,11,12]. Unfortunately, the patients in our series applied to us when they already had persistent azoospermia. They recorded that they had not been sufficiently informed of this topic and had no frozen sperms. With the advances in m-TESE and ICSI, the chances of becoming a father after CT have increased, as observed in our patients and those in other studies [12,14,15]. In studies of heterogeneous patient groups with various types of cancer that were conducted in 2003, 2011, and 2016, sperms were detected using m-TESE in 41.6%, 42.9%, and 37% of the cases, respectively; and in this study, in 34.9% of the cases [13, 16]. Sperms were detected at a rate of 47% using m-TESE and a live birth rate of 27% was achieved in a study similar to ours, with patients aged 19–49 years who had undergone CT/RT due to ALL (21 cases), HL (9 cases), AML (7 cases), NHL (7 cases), rhabdomyosarcoma (7 cases), bladder cancer (3 cases), and osteosarcoma (2 cases), and who did not receive relapse treatment and did not undergo sperm cryopreservation within three years following RT/CT [16]. The difference between our patients and those in other studies was that our patients completed the maintenance and recurrence treatments during their infertility treatment. In the other studies, most of the m-TESE procedures were performed during CT or in the early period after CT (within one to three years) [16]. Our study had a higher success rate in generating a reservoir for spermatogenetic activity [14]. There is certainly a definitive rate of finding sperms after CT. However, the duration of CT sessions, differences in chemotherapeutic agents, maintenance doses, and treatment of recurrences are definitive factors of the success rate of sperm retrieval. Success rates vary significantly considering the m-TESE results of individual cancer patients. Therefore, we believe that the success rates for homogenous groups should be evaluated separately according to the type of CT and the maintenance and recurrence treatment. This approach may be more descriptive before treatment of cancer in patients who require fertility. Regarding individual factors of sperm retrieval, the mean age of the patients who were ontologically diagnosed in our study was 30 years, whereas the mean age in different studies was ≥35 years [13,15,16]. The total pregnancy rate was 20.63% and the baby take-home rate was 12.7% in our study, whereas the total pregnancy rates in other studies were 50% and 34.8, and the baby take-home rates were 42% and 27.3% [13].

Various factors, such as different oncological diagnoses, type of CT administered, maintenance and recurrence therapies, SDF, high oxidative stress, sperm motility or nonmotility, the age of the woman, oocyte maturation, and therefore, embryonic development were considered in monitoring different rates [13,17]. In a study that involved 73 patients with NHL, HL, leukemia, testicular cancer, sarcoma, and neuroblastoma with post-CT azoospermia, the mean FSH (21.9 mUI/mL), TT (354.4 ng/dL), and testicular volumes (9.1/mL) before m-TESE were predictive factors both for m-TESE and ICSI, as in our study [17]. Unlike this study and others, the ROS value before the m-TESE and the presence of SDFI and motile sperms in the pregnancies and live births following ICSI established a different perspective for the patients in our study.

In our study, the sperm detection rate (50.7%) in patients who had undergone CT for testicular cancer was high, as in the other studies (39%–62%) [17,18]. This was probably because platinum-based therapies were low gonadotoxic [18,19]. Our sperm detection rate was low especially in the NHL patients in the lymphoma group and in the ALL patients. These patients had received more gonadotoxic alkylating agents [18,19]. In the study composed of 17 patients, an irreversible azoospermia rate of 58.8% was detected in our study, where the patients received an alkylating agent such as cyclophosphamide. It had been reported that in the presence of these agents, the m-TESE success rates were lower [18,19].

Although the Jhs score was detected differently as 2 and 3, sperms were retrieved from the m-TESE. Thus, it was observed that the spermatogenetic activity maintained its heterogenicity along all seminiferous channels also in the chemotherapeutic persistent infertile azoospermia patients, such as the patients who did not receive cancer treatment, and that pathology was not important for the next m-TESE or empirical medical approaches. Therefore, when performing m-TESE, it is necessary to check all seminiferous channels, as with other azoospermia patients. The duration of our m-TESE procedures was the same as the average of the azoospermia patients who did not receive CT.

A sperm structure that is high in ROS may lead to a problem in the sperm count and even in sperm production and may cause infertility [20]. Oxidative stress reduces fertilization, pregnancy, and live birth rates [20]. Genital tract infections, varicocele, spinal cord injury, diabetes, obesity, tobacco smoking, alcohol use, recreational drug abuse, ionizing radiation, psychological stress, strenuous exercise, air pollutants, and chemotherapeutic agents induce oxidative stress [20]. The high ROS level in our study was considered a reflection on the ejaculate of persistence of the permanent tissue damage that probably developed after CT. Although no other factor affected ROS microbiologically, we still did not think that it was an objective indicator of ROS. However, the success of the m-TESE in our study emerged as a good/bad criterion of the pregnancy and baby take-home rates. The high FSH value in the m-TESE (−) group, the low levels of semen volumes and testicular volumes, and the fact that the same findings were arrived at in the patients who were ROS (+) and had an SDFI were accepted as objective findings of the negative impact of CT. We considered that as the Jhs was 6 in the patients with a normal ROS and FSH and from whom no sperm was retrieved at the m-TESE, the impact of CT on testicular tissue was accurately reflected in ROS and FSH. As a result, although no sperm was retrieved, there was spermatogenetic activity in the tissue. Almost no spermatogenetic activity was detected in the pathology JHS < 4 in the patients who were ROS (+)(>1.42) and had a high FSH.

The SDFI rate is an important criterion of the potential for fertility and the pregnancy rate [21]. In unexplainable fertilization failures, miscarriages, and pregnancy wastages, the SDFI is checked [21]. We know that the sperms in the testes have the best SDFI along the ejaculatory duct [22]. Within this scope, does CT harm sperm DNAs? While the SDFI rate was low in 13 patients, it was >15–30 in four patients. The fact that the SDFI was not high in all the patients showed that CT did not have a completely negative impact on sperm DNAs. The SDFI rates were high in patients with a high ROS. The fact that nonmotility and significant sperm head abnormalities were observed in patients with a high ROS and SDFI showed that ROS and SDFI are important criteria before m-TESE and ICSI.

Time-lapse may be utilized to select the embryos with the highest potential of achieving pregnancy after monitoring the early and late morphological characteristics of the embryos as well as the rate and timing of their development [10]. In multicenter studies, the pregnancy rate was very low in embryos in which the second cleavage (tPB2) occurred five h earlier. In our study, the mean period of the occurrence of the second cleavage was 5 h. In patients with immotile sperms (i.e. HL, NHL, osteosarcoma, and seminoma patients), the cleavage periods were >8 h. The cleavage periods at a time-lapse were prolonged by >12 h especially in patients with a high ROS rate, who were m-TESE (+), and who had an SDFI of >25. Time-lapse was an indicator—although subjective—of the negative impact of CT on the embryo.

Some patients were previously m-TESE (−), but sperms were retrieved from them after repeat m-TESE. They did not receive any treatment intended for fertility. Although the reason for this was not fully understood, possible reasons are the practical knowledge of the clinic where the m-TESE was performed, the application of macro-TESE instead of m-TESE, and very short surgical periods. Thus, the administration of a single m-TESE in patients is insufficient and should be continued for sperm detection.

In conclusion, sperms may be detected using m-TESE in patients who develop persistent azoospermia associated with CT due to different oncological diagnoses. Unlike other studies, our study revealed that a low FSH value and normal ejaculatory ROS rates are positive predictive factors of sperm detection before m-TESE. The motility of the sperms detected after m-TESE and normal SDFI rates were found to be positive predictive criteria of high fertilization, good embryonic cleavage, pregnancy, and live birth.

## Figures and Tables

**Table 1:** Demographic characteristics: oncological diagnoses, body mass index (BMI/kg).

	Min–max	Median	
Patients/years	21–42	30	
Spouse’s age/years	18–36	25	
Duration of marriage/years	0.80–12.60	3.4	
**Oncological diagnoses**	**Number**	**%**	**BMI/kg min–max**
Seminoma	24	38	14.1–22.3
Nonseminoma	8	12.7	14.2–23.2
ALL	8	12.7	10.7–24.1
AML	3	4.8	13.3–17.9
CML	1	1.6	-
Hodgkin’s lymphoma	4	6.3	13.77–21.57
Non-Hodgkin’s lymphoma	8	12.7	15.66–29.46
Rhabdomyosarcoma	2	3.2	-
Soft tissue Ewing sarcoma	1	1.6	-
Osteosarcoma	3	4.8	11.5–28.1
Wilms tumor	1	1.6	23.4
Smoking (−) rate	47	74.6	**-**

SSPS 22.0

**ALL:** Acute lymphoblastic leukemia. **AML:** Acute myelocytic leukemia. **CML**: Chronic myelocytic leukemia. **Johnsen score of testicular biopsies score morphological base:** 1 Tubular sclerosis, 2 Sertoli cells only, 3 Spermatogonia only, 4 Arrest at primary spermatocyte, no, spermatids, 5 Many spermatocytes, no spermatids, 6 No late spermatids, arrest at spermatid stage, 7 No late spermatids, but many early spermatids, 8 Few late spermatids 9 Many late spermatids, disorganized tubular epithelium, 10 Full spermatogenesis

**Table 2:** Number and success rate of micro-testicular sperm extraction, semen reactive oxidative stress, testicular pathological results, DNA fragmentation of sperms in the testicular tissue, pregnancy rates, fertilized oocyte count, and embryo separation period at time-lapse.

	Number	Means/results	*%*
**m-TESE (+) total results**	63	22(+)/41(−)	**34.9**
m-TESE 1^st^	48	16(+)/32(−)	-
m-TESE 2^nd^	15	6(+)/9(−)	-
m-TESE 1^st^ motile(m)/nonmotile(im)	22	13(m)/9(im)	-
m-TESE (+) Jhs score	19	6(4–8)	-
m-TESE (−) Jhs score	44	2(1–6)	-
m-TESE time/min.	25–161	102(100.59 ± 21.1)	-
**ICSI-total pregnancy**	13	13/63	**20.63**
ICSI 1^st^ (m-TESE 1^st^)	16	9(+)/63	14.28
ICSI 2^nd^ (m-TESE 1^st^ and 2^nd^)	13	4(+)/63	6.34
**ICSI total take of baby**	8	8/63	**12.7**
ICSI 1^st^ (m-TESE 1^st^)	16	5(+)/63	7.93
ICSI 2^th^ (m-TESE 1^st^ and 2^nd^)	13	3(+)/63	4.76
Number of children before CT	31	1(1–3)	-
Number of oocytes expose to ICSI	168	69 (fertilization)	41
Total time-lapse divided time/h	168	5(4.4–14)	-
SDFI rate in the m-TESE tissue	17	4(15–30)	-
ROS test/mV/10^6^ sperm/mL	0.35–2.56	-	-

SSPS 22.0

**m-TESE:** Micro-testicular sperm extraction, **CT:** Chemotherapy, **Jhs:** Johnsen’s testicular pathologic score, **ICSI:** Intracytoplasmic sperm injection, **Time-Lapse:** Embryoscope^®^, **SDFI:** Sperm DNA fragmentation index. **Motile/immotile sperm:** (m)/(im).

**Table 3:** Age, smoking status, FSH values before surgery and chemotherapy, hormonal values before surgery, semen volumes, testicular volumes, sexual life, and statistical differences between duration of surgery of patients who were positive/negative at microtesticular sperm extraction.

	m-TESE (−)		m-TESE (+)		
	Mean ± s.s./n-%	Median	Mean ± s.s./n-%	Median	P
Patient’s age	29.6 ± 5.1	29	31.3 ± 4.5	31.5	0.189^t^
Nonsmoker	30	73.20	17	77.30	0.937^x^
Smoker	11	26.80	5	22.70	0.937^x^
	**Min–max**	**Median**	**Min–max**	**Median**	**P**
Marriage/year	1.6–6.8	3.2	1.4–7.0	3.55	0.937^m^
m-TESE/min	89.4–120.6	105	64.6–118	99	0.078^m^
Before CT-FSH	6.5–10.5	8.43	7.4–10.4	9.07	0.873^m^
Before m-TESE-FSH	11.6–25.0	16.89	13.5–20.5	17.08	0.812^m^
**Intergroups changes (m-TESE +/−)**	**P**		**P**		
	0.046		0.028		
**Before m-TESE**	**Mean ± s.s./n-%**	**Median**	**Mean ± s.s./n-%**	**Median**	**P**
LH mIU/mL	8.1–18.5	12.26	8.5–14.9	10.91	0.336^m^
TT pg/mL	2.8–6.6	4.44	2.7–7.3	4.08	0.579^m^
PRL ng/mL	6.2–15.4	9	6.3–14.5	9.75	0.863^m^
Semen volumes	2.4–4.6	3.6	1.7–3.7	2.7	0.007^t^
Right testicular volumes	2.7–7.1	5.5	4.2–7.8	6.4	0.045^m^
Left testicular volumes	1.0–10.0	5.3	4.7–8.3	6.8	0.032^m^

m Mann-Whitney U test/

x2 Chi-Square test/Fisher exact

**m-TESE:** Microtesticular sperm extraction. **CT:** Chemotherapy. **FSH**: Follicle-stimulating hormone. **LH:** Luteinizing hormone. **TT:** Total testosterone. **PRL:** Prolactin. **ROS:** Reactive oxidative stress test.

**Table 4:** Evaluation of microtesticular sperm extraction, FSH, pathology, fertilized oocyte count, pregnancy rates, sperm motility/nonmotility, and separation periods at time-lapse according to the result of the reactive oxidative stress and sperm DNA fragmentation.

	ROS(+), >1.42, n = 5		ROS(−),<1.42, n = 12			SDFI > 15, n = 13		SDFI < 15, n = 5		
	Min–max	Median	Min–max	Median	p	Min–max	Median	Min–max	Median	p
**m-TESE(+)**	1.77–3.17	2.44	0.7–1.36	1.11	0.002^m^	5.81–10.59	17	4.19–11.99	8.5	0.002^m^
**FSH mIU/mL**	1.63–5.97	25.6	4.89–11.09	8.96	0.002^m^	1.08–4.12	22.35	3.87–24.29	12.69	0.048^m^
**Jhs score**	1.63–5.97	3	3.64–7.94	5	0.142^m^	0.9–4.23	2	2.42–6.88	4	0.046^m^
**Fertilized oocyts count**	3.18–5.22	4	2.03–6.87	4	0.774^m^	1.0–3.8	5	3.27–6.73	5	0.294^m^
**Time-lapse < 5h**	3.45–5.27	4.3	3.12–5.12	4.4	0.690^m^	3.09–4.87	4	3.27–4.67	4	0.818^m^
	**Negative n/%**	**Positive n/%**	**Negative n/%**	**Positive n/%**	**p**	**Negative n/%**	**Positive n/%**	**Negative n/%**	**Positive n/%**	**p**
**ICSI-total pregnancy**	1/20.0	4/80.0	3/25.0	9/75.0	0.099x2	3/60.0	2/40.0	2/15.6	11/84.6	1.00x2
	**Inmotile n/%**	**Motile n/%**	**Inmotile n/%**	**Motile n/%**		**Inmotile n/%**	**Motile n/%**	**Inmotile n/%**	**Motile n/%**	
**Sperm motility**	3/25.0	2/40.0	5/41.7	7/58.3	0.619x2	4/80.0	1/20.0	5/38.5	8/61.5	0.818x2

m Mann-Whitney u test/

x2 Chi-square test/Fisher exact

**m-TESE:** Micro-testicular sperm extraction, **CT:** Chemotherapy, **FSH**: Follicle-stimulating hormone, **ROS:** Reactive oxidative stress test, **Jhs:** Johnson’s score, **Time-Lapse:** Embryoscope®, **ICSI:** Intracytoplasmic sperm injection. **Johnsen score of testicular biopsies score morphological base:** 1 Tubular sclerosis, 2 Sertoli cells only, 3 Spermatogonia only, 4 Arrest at primary spermatocyte, no, spermatids, 5 Many spermatocytes, no spermatids, 6 No late spermatids, arrest at spermatid stage, 7 No late spermatids, but many early spermatids, 8 Few late spermatids, 9 Many late spermatids, disorganized tubular epithelium, 10 Full spermatogenesis.

**Table 5:** Testicular volumes before and after chemotherapy, semen analysis, hormone, and reactive oxidative stress mean values according to cancer diagnosis.

Oncologıcal diagnoses	Num.	TV/mL before CT/	TV/mL after CT	P	SV/mL before CT	SV/mL after CT	P	SC/mL before CT	SC/mL after CT	P	FSH IU/mL before CT	FSH IU/mL after CT	P	TT pg/mL before CT	TT pg/mL before CT	P	ROS before CT	ROS after CT	P
**Seminoma**	24	49 ± 22	39.6 ± 17.7	0.001^m^	4 ± 1.2	3.6 ± 1.9	0.056^m^	7.9 ± 2.5 × 10^6^	azospermia	0.001^m^	7.89 ± 2.45	18.3 ± 4.87	0.001^m^	12.78 ± 2.89	6.8 ± 3.67	0.001^m^	1.23 ± 0.56	1.77 ± 1.2	0.000^m^
**Nonseminoma**	8	39.89 ± 11.23	29.78 ± 9.2	0.001^m^	3.6 ± 1.1	4.2 ± 1.8	0.126^m^	5.8 ± 3.5 × 10^6^	azospermia	0.001^m^	8.75 ± 3.45	22.34 ± 7.68	0.001^m^	22.78 ± 2	2.78 ± 0.68	0.001^m^	1.45 ± 0.66	1.5 ± 0.34	0.126^m^
**ALL**	8	33.56 ± 22	34.56 ± 6.78	0.023^m^	3.2 ± 1.2	2.3 ± 1.1	0.001^m^	none	azospermia	-	none	23.12 ± 9.87		32.78 ± 12.91	4.8 ± 0.6	0.001^m^	none	1.42 ± 0.34	-
**AML**	3	26.78 ± 3.45	23.45 ± 4.56	0.037^m^	6 ± 2.4	2.2 ± 0.6	0.001^m^	18.79 ± 4.53 × 10^6^	azospermia	0.001^m^	6.57 ± 3.4	18.97 ± 5.67	0.001^m^	21.78 ± 1.8	7.8 ± 3.70	0.001^m^	1.23 ± 0.22	1.34 ± 0.33	0.001^m^
**CML**	1	44.5 ± 13.4	32.44	0.001^m^	4.2	3.3	0.000^m^	none	azospermia		7.89	16.78	0.001^m^	20.78	8.7	0.001^m^	none	1.42	-
**Hodgkin’s lymphoma**	4	33.45 ± 12.34	32.56 ± 11	0.146^m^	4.4 ± 2.1	3.9 ± 1.7	0.107^m^	18.4 ± 4.56 × 10^6^	azospermia	0.001^m^	7.89 ± 2.3	32 ± 13.27	0.001^m^	9.78 ± 2	5.68 ± 3.55	0.001^m^	1.11 ± 0.23	2.34 ± 1.2	0.001^m^
**Non-Hodgkin’ lymphoma**	8	42.34 ± 9.89	40.7 ± 8.79	0.073^m^	3 ± 1.8	2.9 ± 1	0.122^m^	17.4 ± 5.46 × 10^6^	azospermia	0.001^m^	4.56 ± 2	29.87 ± 12.65	0.001^m^	11.78 ± 3.95	6.98 ± 3.73	0.001^m^	0.98 ± 0.22	1.98 ± 0.23	0.001^m^
**Rhabdomyosarcoma**	2	34.67 ± 8.97	32.45 ± 21	0.126^m^	3.4 ± 2	2.9 ± 0.9	0.000^m^	19.67 ± 3.56 × 10^6^	azospermia	0.001^m^	6.45 ± 3.2	18.98 ± 4.67	0.001^m^	12.78 ± 1.96	5.78 ± 2.22	0.001^m^	1.22 ± 0.23	2.31 ± 0.45	0.000^m^
**Soft tissue Ewing sarcoma**	1	none	32.12	-	3.2	2.8	0.026^m^	none	azospermia	-	none	34.2	-	none	4.88 ± 1.75	-	none	1.76	-
**Osteosarcoma**	3	none	45.6 ± 2.1	-	4.4 ± 1.2	3.4 ± 2	0.000^m^	23.34 ± 5.67 × 10^6^	azospermia	0.001^m^	6.78 ± 2.34	15.67 ± 3.78	0.001^m^	none	3.78 ± 1.76	-	1.34 ± 0.23	2.0 ± 1.22	0.000^m^
**Wilms tumor**	1	none	34.5	*-*	3.1	2	0.000^m^	none	azospermia	*-*	none	23.67	*-*	none	7.68 ± 3.77	*-*	none	1.14	*-*

m Mann-Whitney U test

**ALL:** Acute lymphoblastic leukemia. **AML:** Acute myelocytic leukemia. **CML**: Chronic myelocytic leukemia. **FSH:** Follicle-stimulating hormone. **ROS:** Reactive oxidative stress test. **CT:** Chemotherapy, **TT:** Total testosterone
